# Optimization of *Stryphnodendron adstringens* (Barbatimão) Extraction: Chemical Evaluation, Cytotoxicity, Antioxidant and Anti-Inflammatory Activities

**DOI:** 10.3390/molecules31020224

**Published:** 2026-01-09

**Authors:** Cynthia Nara Pereira de Oliveira, Thainá Gomes Peixoto, Luiz Gustavo Modesto Lobo Teixeira, Samuel Beiral Alves Pessoa, Nicole Maia Pedrosa, Viviane Flores Xavier, Paula Melo de Abreu Vieira, Cristina Duarte Vianna Soares, André Augusto Gomes Faraco, Karina Barbosa de Queiroz, Fernanda Guimarães Drummond e Silva, Rachel Oliveira Castilho

**Affiliations:** 1 Department of Pharmaceutical Products, Faculty of Pharmacy, Federal University of Minas Gerais (UFMG), Belo Horizonte 31270-901, Brazil; cynthianara@gmail.com (C.N.P.d.O.); luizteixeira1209@ufmg.br (L.G.M.L.T.); samuel.bap9405@gmail.com (S.B.A.P.); nicolemaia.pedrosa00@icloud.com (N.M.P.); cviannas@yahoo.com (C.D.V.S.); andrefaraco@farmacia.ufmg.br (A.A.G.F.); 2 Postgraduate Program in Health and Nutrition, Federal University of Ouro Preto (UFOP), Ouro Preto 35402-145, Brazil; thaina.peixoto@ufop.edu.br (T.G.P.); karina.queiroz@ufop.edu.br (K.B.d.Q.); 3 Morphopathology Laboratory, Center for Research in Biological Sciences, Federal University of Ouro Preto (UFOP), Ouro Preto 35402-136, Brazil; viviane.xavier@aluno.ufop.edu.br (V.F.X.); paula@ufop.edu.br (P.M.d.A.V.); 4 Food Department, Nutrition School, Federal University of Ouro Preto (UFOP), Ouro Preto 35402-145, Brazil; 5 Brazilian Academic Consortium for Integrative Health (CABSIN), Rio de Janeiro 05449-070, Brazil

**Keywords:** extraction optimization, *Stryphnodendron adstringens*, barbatimão, chemical evaluation, cytotoxicity, antioxidant activity, anti-inflammatory activity

## Abstract

Extracts from the stem bark of *Stryphnodendron adstringens* (barbatimão) exhibit relevant medicinal properties, such as anti-inflammatory, antioxidant, antimicrobial, and wound-healing activities, which reinforce their potential for developing herbal medicines. The $550 billion plant bioactive market (by 2030) demands safer, green-chemistry-aligned extraction methods for responsible industrial scaling. In this study, dry extracts obtained from the stem bark of *S. adstringens* were obtained by ultrasound-assisted maceration in one- and two-step extraction systems. Parameters such as yield, solvent evaporation time, cost, acute toxicity, epigallocatechin gallate (EGCG) concentration, cell viability, antioxidant potential, and anti-inflammatory activity were evaluated. High-EGCG two-step organic extracts were industrially difficult, needing more raw material and toxic solvents. In contrast, the single-step extracts showed a better balance between yield, cost, safety, and biological efficacy. All extracts showed cell viability above 70% at safe concentrations and significantly reduced the production of inflammatory cytokines. Thus, the results confirm that optimizing single-step extraction, with lower environmental impact solvents, enables producing safe and effective polyphenol-rich extracts, consolidating water as the main candidate for industrial-scale phytotherapeutic formulations of barbatimão, in line with its traditional use in infusions.

## 1. Introduction

*Stryphnodendron adstringens* (Mart.) Coville, popularly known as barbatimão, is a native species from Brazil, particularly in the Caatinga and Cerrado biomes [1]. This plant was used in ancient times by the Brazilian indigenous: “The bark is strongly astringent, known as the “bark of youth” or “bark of virginity”, and its energetic styptic action is positive in antileukocorrhea and antidiarrheal, hemostatic or paralyzing for hemoptysis and uterine hemorrhages and also combats scurvy conditions, hernias, impetigo, wounds and ulcers, and is most widely used in the tanning industry” [2].

*S. adstringens*, stem bark, is rich in condensed B-type tannins, proanthocynadin, prodelphinidins, prorobinetidinins, and profisetenidin (e.g., (epi)gallocatechin, 4′-O-methyl-gallocatechin, and (epi)gallocatechin-3-O-gallate), in addition to the monomeric unit of hydrolyzable tannins, gallic acid [3,4]. The large presence of tannins in barbatimão species is related to its medical potential, with healing, anti-inflammatory, antioxidant, antimicrobial, and antiulcerogenic activities demonstrated in multiple in vitro and in vivo tests [5].

The antioxidant activity of phenolics is one of the main bioactivities explored and is widely documented [6,7,8]. Phenolics can: neutralize and eliminate free radicals by donating a hydrogen atom from their hydroxyl group to the radical; chelate metals that participate in radical generation; reduce α-tocopheryl radicals; inhibit oxidases; increase uric acid [9]; inhibit ROS-producing enzymes; transfer electrons to stabilize reactive species; and stimulate increased synthesis of antioxidant enzymes [7,9,10,11]. The anti-inflammatory activity of tannins is due to the inhibition of pro-inflammatory mediators, such as COX (Cyclooxygenase); the stimulation of Nrf2 (nuclear factor erythroid 2); the inhibition of NFκB (nuclear factor kappa B) activation; and, consequently, the production of inflammatory cytokines. The excessive production of IL-1β (interleukin 1 beta), IL-6 (interleukin 6), and TNF-α (tumor necrosis factor alpha) is generally associated with the development of various diseases, and phenolics have demonstrated the ability to inhibit them [11,12]. In addition, phenolics can influence the differentiation of cells related to the immune system [11]. In a study by Koch (2019) [7], epigallocatechin gallate was able to reduce Th1 and Th17 differentiation. Furthermore, it can act as a free radical scavenger by both hydrogen ion donation and electron transfer, in addition to inhibiting nitric oxide synthase (iNOS) and COX-2 [13].

Barbatimão is included in official Brazilian compendia, such as the Brazilian Pharmacopoeia, the Phytotherapeutic Memento, and the Brazilian Pharmacopoeia Phytotherapeutic Formulary because of its pharmaceutical pharmaceutical interesting properties, and assessment through the Unified Health System (SUS) under preparations with healing properties. The recommended dosage forms are infusion of the bark or ointment made from the aqueous extract [14,15,16].

Developing and industrializing herbal medicines initially involves research into the chemical composition, selection of chemical markers, development of analytical methods, and obtaining extracts with potential bioactivity, the way to quality control of the finished product. Thus barbatimão’ tannin-rich extracts are usually obtained in water, hydroalcoholic, and acetone:water solvents. Propylene: water can also be used as solvent for some pharmaceutical formulations [4].

The rational choice of the solvent extraction system is a key step in establishing the chemical matrix and the final yield of the process, as it aids in separating substances of interest in complex matrices, such as plant materials. Pure organic solvents, as well as combinations of them, are widely used in the extraction processes of compounds of interest from plant materials. Although very useful in these separations, organic solvents have many disadvantages, such as high toxicity and high cost. These drawbacks become especially relevant when considering the global herbal medicine market: USD 83 billion, reaching USD 550 billion in 2030 at a compound annual growth rate of 18.9% until 2030 [17].

However, a growing popularity of preparations containing medicinal plants reinforces the need to develop safe and effective herbal compositions [17], free from any toxic solvent residues, whether for application to injured tissue requiring healing and inflammation control, or any other target biological tissue. Similarly, the safety and impacts of industrial scaling processes that use organic solvents on society, the environment, and the global economy must also be discussed and considered.

In studies with barbatimão, our research group developed and validated an analytical method for quantifying chemical markers such as gallic acid, gallocatechin, and epigallocatechin-3-O-gallate (low molecular weight monomers) [18]. This method required sample preparation to separate low- and high-molecular-weight tannins. Therefore, the alcoholic extract was purified by partitioning it into a solvent system called “anti-tannin”, composed of ethyl acetate:*n*-butanol:2-propanol:water (3.5:0.5:1.0:4.5), to separate the different fractions. The two fractions, aqueous and organic, rich in high- and low-molecular-weight tannins, respectively, were subsequently evaluated for their antiparasitic, anti-inflammatory, and antioxidant activities, demonstrating that the organic fraction, rich in low-molecular-weight tannins, was more active [3,18,19]. However, taking into account that ethyl acetate, *n*-butanol, and 2-propanol are toxic solvents and should not be used in pharmaceutical formulations, the objective of this work is to optimize the process of obtaining barbatimão extract and preserve its intrinsic biological activities, while aligning the principles of green chemistry and the viability of scaling for industrial applications.

## 2. Results and Discussion

The organic fraction obtained by partitioning with the mixture of ethyl acetate:isopropanol:*n*-butanol (0.7:0.2:0.1) solvents in previous studies showed promise in evaluating antiparasitic, anti-inflammatory, and non-cytotoxic activity [3,18,19]. However, taking into account that this solvent system is toxic and the preparation process is long, the extraction was optimized to obtain barbatimão extract with the same activities of the organic fraction, while also aligning the principles of green chemistry, with less toxic waste, greater operational safety and products, and finally greater scalability viability for industrial applications.

Therefore, different *S. adstringens* extracts are obtained in a single extract step for comparison with the previous process in two steps. The extraction method used was maceration assisted by ultrasonication, using different types of solvents and solvent mixtures, as presented in Table 1. The solvents used in the extraction in a single step were:ethyl acetate, acetone, acetonitrile, water, dichloromethane, ethanol, water:acetone (1:1), water:ethanol (1:1) and ethyl acetate:isopropanol:*n*-butanol (0.7:0.2:0.1). In turn, ethanolic extract and then liquid–liquid partition with a mixture of ethyl acetate:isopropanol:*n*-butanol (0.7:0.2:0.1) solvents was used to extract in two steps in order to compare with results obtained in previous works. Therefore, this liquid–liquid extraction by partition in two steps, results in two different extracts: the aqueous phase and the organic phase of the extraction. Many solvents used in this study are not considered part of green chemistry, such as ethyl acetate, acetonitrile and dichloromethane, but are used for comparison of the chemical matrix, since the starting point was the organic fraction of the extraction in two steps. The extracts obtained were then analyzed by HPLC-DAD (high-performance liquid chromatography (HPLC) coupled to a diode array detector) in the method developed by Nascimento et al. (2013) [18] for quantifying chemical markers in barbatimão extracts.

The characteristics and properties were selected considering the most relevant parameters for the industrial scalability of the extraction process. These include yield, solvent evaporation time, cost, acute oral toxicity of the solvent or mixture (LD_50_ in mg/kg of carbon), and commercial control by the Brazilian Federal Police.

The extractive yields in Table 1 ranged from 2.0 mg to 671.04 mg per gram of dry starting plant material in g. The time elapsed to completely evaporate the solvents ranged from 0 to 110 min. The LD_50_ values, referring to the acute toxicity of the solvents or solvent mixtures, ranged from 1600 to 10,000 mg of the substance per kg of mouse. The results in Table 1 show that the aqueous extract (10) and the organic extract (11), obtained in two steps, consume a greater mass of plant material and take longer to obtain; they have, 25 g of material as a starting point, with an ethanolic extract initially being obtained and then aqueous and organic extracts after a second extraction (ethyl acetate: *n*-butanol:2-propanol: water). In this case, the organic extract (11) has the best yield in terms of mass when compared to the aqueous extract (10). When observing the results of the extracts obtained in a single step, or direct extraction, those which showed the highest yield, (and also taking into account that they used less starting plant material of (5 g), were the aqueous extract (4), followed by the acetone: water (1:1) (7), ethanol (6) and ethanol: water (1:1) (8).

On the other hand, aqueous extracts take longer to eliminate the solvent when compared with organic extracts such as ethyl acetate, acetone, acetonitrile, and ethanol. Water elimination makes the process slower and more technologically expensive, since dry extracts are superior for herbal medicine production due to their physical, chemical, and microbiological stability, as well as the ease of standardizing active ingredients, making them more viable for large-scale production. Techniques such as nebulization (spray-drying), spouted bed drying, freeze-drying, and rotary evaporation are used to prepare dry extracts [20]. The ideal choice is equipment with the greatest potential for drying extractive solutions from medicinal plants, ensuring a high-quality final product at a low investment. However, spray dryers are typically used for drying liquid extracts in industry [21]. In terms of security and control by the Brazilian federal police, aqueous extract is the best extraction solvent when compared to organic solvents such as acetonitrile, dichloromethane, butanol, and propanol.

Water also stands out in terms of solvent production costs. The cost of solvents or solvent mixtures per liter ranged from USD 0.03 to USD 26.94. Only 2 of all the 8 different solvents tested do not have their commercialization controlled by the Brazilian Federal Police.

### 2.1. Phytochemical Characterization of S. adstringens Extracts by HPLC-DAD

**Figure 1:**
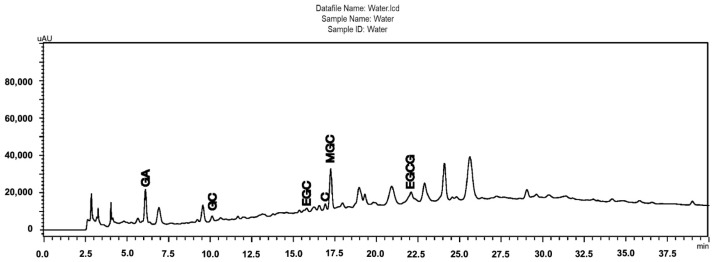
Chromatographic profiles obtained by HPLC-DA of the *Stryphnodendron adstringens* (Barbatimão) aqueous stem bark extract obtained by a one-step extraction process, containing the majority of the identified peaks (GA, gallic acid; GC, gallocatechin; EGC, epigallocatechin; C, catechin; EGCG, epigallocatechin gallate; MGC, 4′-O-Methylgallocatechin); Chromatographic conditions: pre-column C18 (XDB Zorbax^®^, 4 × 4 mm I.D.; 5 μm), attached to a C18 column (LiChrospher100, 250 × 4 mm I.D.; 5 μm); linear gradient elution, A–B (95:5% [*v*/*v*]) to A–B (60:40% [*v*/*v*]) in 60 min with 0.1% phosphoric acid (A) and 0.1% phosphoric acid in acetonitrile; 1 mL/min; 4 °C; 10 mL; l = 210 nm.

Figure 1 shows the chromatographic profile of the *S. adstringens* aqueous stem bark extract obtained by a one-step extraction process [18]. As can be seen highlighted in the chromatogram, the aqueous extract contains gallic acid (GA), gallocatechin (GC), epigallocatechin (EGC), catechin (C), epigallocatechin gallate (EGCG), and 4′-O-Methylgallocatechin (MGC). Phytochemical characterization was performed by Ultra Performance Liquid Chromatography (UPLC) coupled to Electrospray Ionization Mass Spectrometry (ESI-MS). The main compounds were identified through analysis of UV spectral data and ESI-MS fragmentation patterns. This identification was validated by comparison with reference standards or with data reported in the literature for *S. adstringens*. The species has a chemical composition rich in flavan-3-ol oligomers, which include prodelphinidins, prorobinetinidins and other related derivatives [3]. All other extracts obtained were also evaluated using the same method and it was possible to observe the same chemical matrix, including the substances identified in the chromatogram in Figure 1, but with different concentrations according to the peak areas (Supplementary Material). 

**Figure 2:**
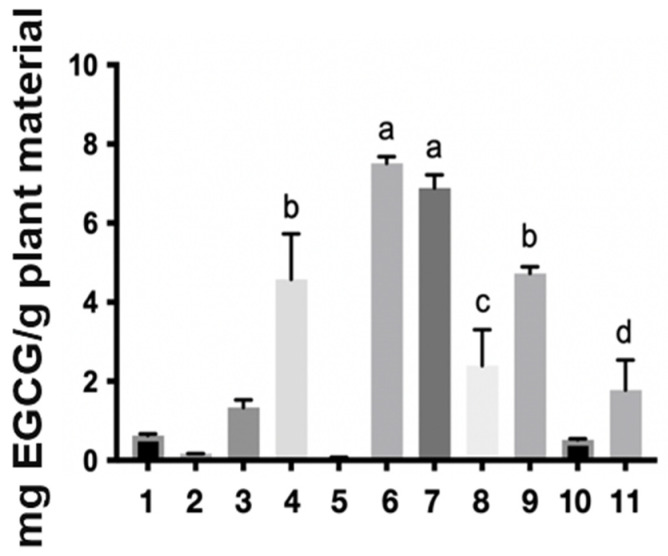
Extractive yield in milligrams of epigallocatechin gallate per gram of plant material in the different *Stryphnodendron adstringens* extracts. The numbers represent the extracts: ethyl acetate (1), acetone (2), acetonitrile (3), water (4) dichloromethane (5), ethanol (6), water:acetone (1:1) (7), water:ethanol (1:1) (8) and ethyl acetate:isopropanol:*n*-butanol (0.7:0.2:0.1) (9), two-step water (10), two-step ethyl acetate:isopropanol:*n*-butanol (0.7:0.2:0.1) (11). The letters a, b, c and d in the figure indicate statistical differences. The same letter indicates that there is no significant difference.

Regarding the levels of the reference markers, the epigallocatechin gallate (EGCG) content found in the samples ranged from 0.08 to 25.82 mg per gram of starting plant material (Table 1 and Figure 2). The ethanol extract (6) stood out in the one-step extraction methods with 7.51 mg/g, water: acetone (7) with 6.89 mg/g, solvent mixture (9) with 4.73 mg/g, and water (4) 4.58 mg/g. In turn, the aqueous extract (10) showed a yield of 7.80 mg/g in the two-step method, while the organic extract (11) presented 25.82 mg/g. These results corroborate the mass/mass yield of the extracts, in which the acetone: water (1:1) (7), ethanol (6), and aqueous (4) extracts also stood out. It is worth noting that the two-step methods showed the best yields of the chemical marker 7.80 and 25.82 for the aqueous and organic extracts, respectively, with the organic extract having a high concentration of the marker, which is likely related to its anti-inflammatory activity [3]. However, obtaining this extract requires a greater mass of plant material, more preparation time, and the use of toxic organic solvents, as expected, making this input unviable for the industrial production of herbal products based on *S. adstringens*.

The analysis of the results (Table 1 and Figure 2) clearly demonstrates the feasibility of reducing the extraction process from two steps to just one, without compromising efficiency, cost, or toxicity. The solvents that performed best across all parameters analyzed were: ethanol (6), water:acetone (7), water (4), and single-step mixtures of solvent (9). The success of the mixture of water with protic (ethanol) and aprotic (acetone) solvents is due to water’s ability to interact with and solvate polyphenols, as widely reported in the literature [22]. Furthermore, factors such as evaporation time, acute toxicity, and commercial control (Brazilian Federal Police) are crucial parameters that directly impact the industrial scalability of these processes.

Given these results, the antioxidant activity was evaluated in three different methods: Folin–Ciocalteau reagent reducing substances (FCRRS); Ferric Reducing Antioxidant Power (FRAP); and, Oxygen radical absorbance capacity (ORAC).

### 2.2. Antioxidant Activity of S. adstringens Extracts

**Figure 3:**
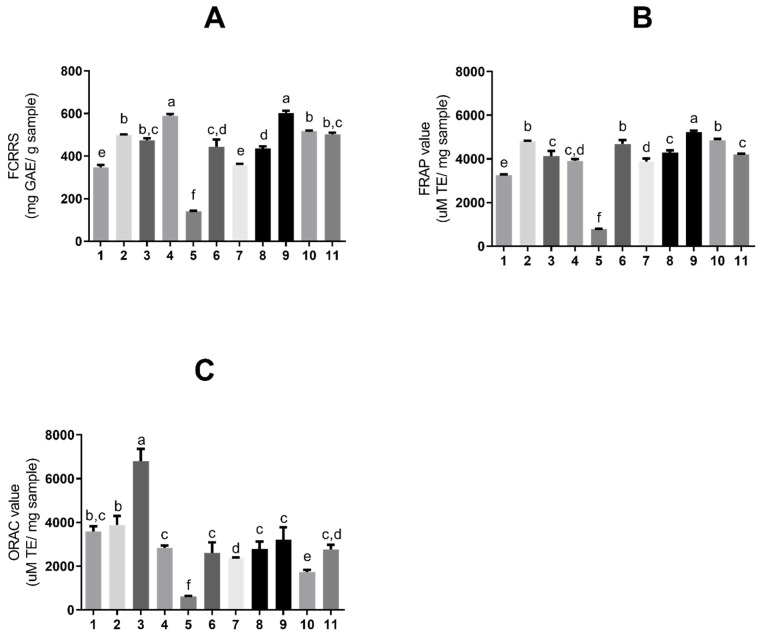
Antioxidant activity expressed as (**A**) total reducing substances by Folin–Ciocalteu, (**B**) ferric reducing antioxidant power (FRAP), and (**C**) oxygen radical absorbance capacity (ORAC), for extracts and fractions of *Stryphnodendron adstringens* extracts and fractions. Results are presented as means ± standard deviation. Bars with different letters differ from each other by one-way ANOVA, followed by Tukey’s post hoc test (*p* < 0.05). GAE = gallic acid equivalents; TE = Trolox equivalents. The numbers represent the extracts: ethyl acetate (1); acetone (2); acetonitrile (3); aqueous (4); dichloromethane (5); ethanol (6); aqueous/acetone (7); aqueous/ethanol (8); solvent mixture (9); aqueous fraction (10) and organic fraction (11).

According to well-documented scientific publications, tannins exhibit strong antioxidant activity. Therefore, assays were conducted to assess the antioxidant activity of the obtained extract solutions. Figure 3 shows the antioxidant activity of *S. adstringens* extracts and fractions, respectively, determined by the Folin–Ciocalteu (6A), FRAP (6B), and ORAC (6C) methods. The Folin–Ciocalteu reducing capacity (FCRRS assay) is based on the substance’s ability to transfer electrons in an alkaline medium. Although it is used to determine total phenolics, it is not a specific method and quantifies reducing substances in general, such as vitamin C [23,24].

Hence, in this study we considered the assay as indicating the total reducing-substance content of the Folin–Ciocalteu reagent. Similarly, the FRAP assay is based on electron transfer, assessing the sample’s ability to reduce ferric ion (Fe^3+^, complex with tripyridyltriazine—TPTZ) to ferrous iron (Fe^2+^), which increases absorbance. The ORAC assay is based on hydrogen-donating capacity and represents a competitive method that best mimics physiological mechanisms [24]. Thus, a combined analysis of the results obtained by the different methods allows for a more in-depth understanding of the antioxidant activity of each extract.

The assays shown in Figure 3A,B are based on electron donation [24]; thus, there are noted similarities between the extracts with higher values in both. The extract with the greatest reducing capacity (Figure 3A) was a one-phase solvent mixture (9) (602.67 ± 10.08 mg GAE/g sample) and aqueous (4) (589.46 ± 7.93 mg GAE/g sample), also in one phase.

The extract with the highest activity in FRAP (Figure 3B) was a one-phase solvent mixture (9) (5226.89 ± 71.29 μM TE/mg sample). The extracts with the highest ORAC value (Figure 3C), an assay based on hydrogen ions, were acetonitrile (3) in one phase (6809.51 ± 556.5 μM TE/mg sample). Table 1 summarizes all the results obtained for the extraction assays and antioxidants.

The difference between the extracts with the highest FCRRS and FRAP values relative to ORAC indicates that the mechanism of action of the phenolic compounds in these samples is mediated by electron donation to the radical rather than by hydrogen ion transfer.

The samples with the highest values in Folin–Ciocalteu and FRAP in a mixture of solvents and aqueous were the second highest in EGCG concentration per gram of plant material (Table 1). This compound has four aromatic rings and eight hydroxyl groups, which confer high antioxidant capacity through radical scavenging and contribute to the total antioxidant capacity.

The elevated increase in the number of hydroxyl groups enables the structure to act by capturing electrons and scavenging radicals [25]. In fact, the location and number of hydroxyl groups on the ring confer greater antioxidant properties compared with structurally related molecules, such as epigallocatechin (EGC) or epicatechin (EC), as well as confer greater water solubility. Moreover, variation in the EGCG structure (i.e., the ortho-3′,4′-dihydroxy portion and the 4-keto, 3-hydroxyl or 4-keto, 5-hydroxyl portion) enables metal ion chelation [13,26]. The higher water solubility may explain the greater levels of this compound in extracts obtained with polar solvents or solvent mixtures. However, it is likely that the presence of other antioxidant compounds has influenced the values obtained for these samples, since samples with the highest EGCG concentration do not appear among the higher FCRRS and FRAP values.

The sample chromatogram obtained with a one-phase solvent mixture indicates extraction of hydrophilic compounds with low molecular weight and hydrophobic compounds with medium molecular weight, occurring between 15 and 30 min. In turn, the aqueous extract (one phase) shows peaks that indicate the extraction of less polar tannins. Thus, it can be inferred that the compounds responsible for the antioxidant activity in these samples are less polar compared with the water–ethanol extract, since the extraction of this system reduced the presence of these compounds, and the sample did not perform well in terms of antioxidant activity.

Gallic acid, a phenolic acid precursor of hydrolysable tannins, has a lower molecular weight and higher polarity compared with other phenolic compounds [27,28]; therefore, extractive systems with higher polarity extract it more efficiently than others.

Although the number of studies is limited, using polar solvents to obtain the best extraction of barbartimão bark is common and is associated with better antioxidant results compared with extracts obtained with nonpolar solvents, such as chloroform [4].

The acetonitrile extract also does not have the highest EGCG concentration; nonetheless, the chromatogram indicates the most intense extraction of compounds overall, mainly medium-polarity compounds, occurring between 15 and 30 min. Consequently, it is possible that the solvents could extract less polar tannins, which act by hydrogen ion donation.

A study on *S. adstringens* species showed that antioxidant protection levels were higher by ion reduction than by OH radical scavenging [29]. The reducing capacity of *S. adstringens* extracts can prevent radical production by the Fenton reaction, in which free iron acts as a catalyst in a reaction that generates superoxide and hydroxyl radicals [29].

In fact, these results were anticipated due to the *S. adstringens* composition, which is mainly hydrolysable tannins such as gallic acid, and condensed tannins such as proanthocyanidins, which are monomers of flavan-3-ols such as gallocatechin and epigallocatechin gallate [29,30] with two phenolic rings [31], potentially conferring high antioxidant capacity.

Gallic acid, which influenced electron transfer capacity in the present study, also acts through the Keap1/Nrf2/ARE pathway, enabling translocation of Nrf2 to the nucleus and consequent transcription of antioxidant enzymes. The tannins present in *S. adstringens* bark can scavenge free radicals and inhibit Fe^2+^-induced lipid peroxidation [30].

Nevertheless, tannins can interact with proteins, denaturing them. This ability enables reducing reactive oxygen species, an anti-mutagenic effect, and antiviral and antimicrobial activities [31].

Some extracts exhibit significantly higher antioxidant assay values than available standards (Supplementary Material). The aqueous extract and the solvent mixture, both in one phase, resembled catechin in the FCRRS, while all samples in FRAP showed results like this standard, except for the dichloromethane extract. Extracts obtained with acetone, ethyl acetate, a solvent mixture, and the one-phase organic fraction resembled EGCG in the ORAC assay. The aqueous extract showed activity like catechin, while water–acetone, water–ethanol, and ethanol (all in one phase) resembled gallocatechin.

In analyzing all the results shown in Table 1, the extractive systems which concurrently achieved better performance, higher CRS (Chemical Reference Substance) concentration, shorter solvent evaporation time, lower cost, lower toxicity, and higher antioxidant activity were the one-phase extraction systems: water, water–ethanol, acetone, acetonitrile, ethanol, solvent mixture, aqueous fraction, organic fraction, ethyl acetate, and water–acetone.

### 2.3. Sulforhodamine B Colorimetric Assay for Cytotoxicity of S. adstringens Extracts

The anti-inflammatory activity of tannins is already well described in the literature. In general, phenolic compounds such as tannins can reduce inflammation by decreasing oxidative stress and suppressing pro-inflammatory signaling pathways, which, in turn, limits the production of inflammatory molecules such as nitric oxide. This dual action helps protect against several chronic diseases that are aggravated by both oxidative stress and low-grade inflammation. Since extracts from the stem bark of *S. adstringens* showed antioxidant activity, in vitro assays were conducted to evaluate whether these extracts also exhibited anti-inflammatory activity.

**Figure 4:**
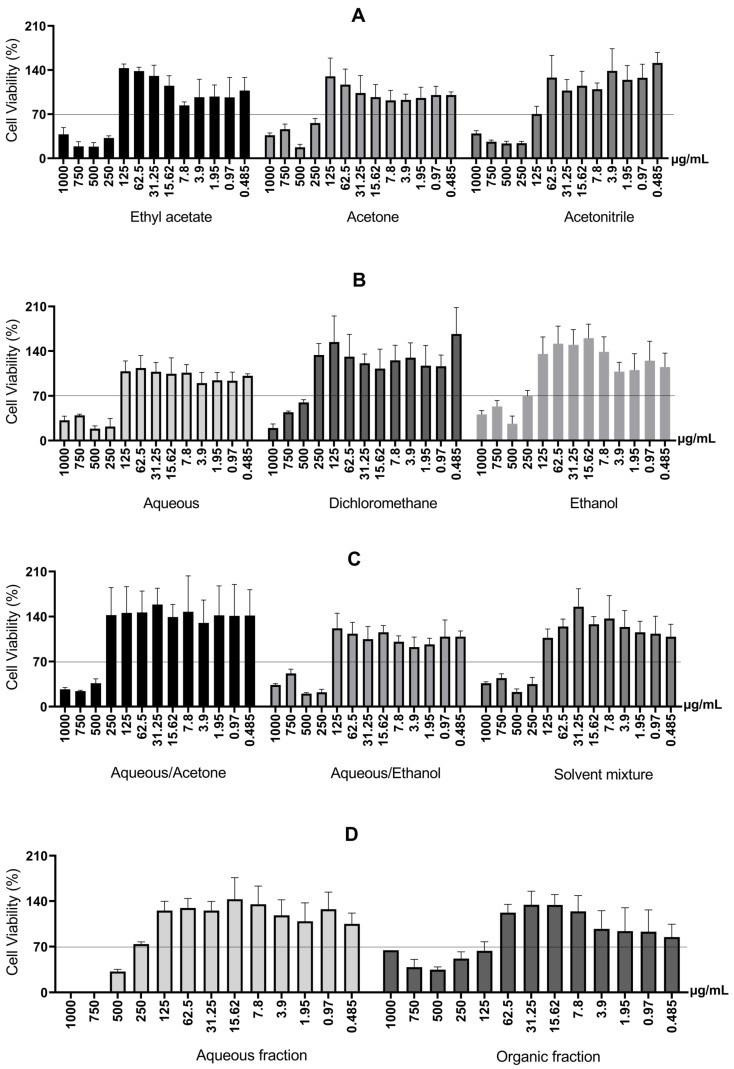
Cell viability of RAW 264.7 macrophage cells after 24 h, for different extractive systems. (**A**) Cell viability of RAW 264.7 macrophage cells treated with *S. adstringens* stem bark extract obtained using Ethyl acetate, Acetone, Acetonitrile. (**B**) Cell viability of RAW 264.7 macrophage treated with *S. adstringens* stem bark extract obtained using aqueous, dichloromethane, ethanol. (**C**) Cell viability of RAW 264.7 macrophage cells treated with *S. adstringens* stem bark extract obtained using aqueous/acetone, aqueous/ethanol, solvent mixture. (**D**) Cell viability of RAW 264.7 macrophage treated with *S. adstringens* stem bark extract obtained using aqueous fraction and organic fraction. Values expressed as mean of quadruplicates ± standard deviation. The percentage is relative to untreated cells. The minimum viability observed was 70%. These results are representative of two independent assays.

First, the cell viability of RAW 264.7 macrophage cells was measured after 24 h in a monolayer culture to screen the cytotoxicity of barbatimão bark extracts obtained through different extractive systems. The concentration range was 0.485 μg/mL to 1000 μg/mL. The results are presented in Figure 4.

Tannins have distinct molecular structures that affect their polarity and their interaction with the cell surface and the culture medium [32]. Indeed, several studies have reported that tannins with higher molecular weight, higher polymerization, and the presence of galloyl groups are more toxic, including microorganisms [33].

Therefore, the observed increase in cytotoxicity with increasing concentration can be explained by the greater presence of higher molecular weight tannins solubilized in the culture medium. In addition, two-phase extractions involving liquid–liquid partition (mixtures of solvents, aqueous fraction, and organic fraction) use solvents such as alcohols (ethanol, methanol) and acetone with water, which poses the disadvantage of potential residual toxic solvents at the end of the process [6]. The organic fraction and the acetonitrile extract were toxic at 125 μg/mL.

### 2.4. Production of Inflammatory Mediators of Extracts from S. adstringens

Inflammatory activity was measured through the production of nitric oxide (NO) and cytokines induced by lipopolysaccharide (LPS) and interferon-gamma (IFN-γ) in RAW 264.7 cells. LPS is an endotoxin found in Gram-negative bacteria and acts as a Toll-like receptor 4 (TLR-4) agonist. Stimulation of TLR-4 initiates a signaling cascade that mobilizes the transcription factor nuclear factor kappa B (NF-κB), leading to NO, TNF-α, IL-1β, and IL-6 production [34]. On the other hand, IFN-γ belongs to a proinflammatory cytokine family and stimulates the production of other inflammatory biomolecules [35]. Thus, evaluating inflammatory activity through these biomolecular stimulations is reproducible and provides an adequate framework.

Cell viability was required to be at least 70% relative to the control [36] to analyze cytotoxicity. As noted above (Figure 4), all extracts showed cell viability greater than 70% at a concentration of 125 μg/mL or lower.

Although NO is a reactive species, it is considered an inflammatory biomarker because its production begins early in the inflammatory cascade and promotes inducible nitric oxide synthase (iNOS) expression and consequently more NO [34]. Furthermore, NO can stimulate NF-κB, contributing to maintaining the immune response and persistence of the inflammatory stimulus [37].

**Figure 5:**
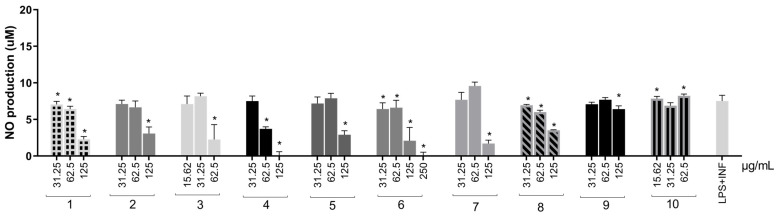
NO production in RAW264.7 cells stimulated by LPS + IFN-γ and treated with *S. adstringens* stem bark extracts. Cells were stimulated for 24 h with LPS + INF-γ and treated with different *S. adstringens* stem bark extracts at the indicated concentrations. The amount of NO released into the culture supernatants is expressed as nitrite. Values expressed as mean of quadruplicates and ± standard deviation (SD). Values marked with * are significantly different (* *p* < 0.005) compared to INF-γ + LPS-stimulated cells without sample treatment by one-way ANOVA followed by Bonferroni post hoc test. These results are representative of two independent assays. The numbers represent the extracts: ethyl acetate (1); acetone (2); acetonitrile (3); aqueous (4); ethanol (5); aqueous/acetone (6); aqueous/ethanol (7); solvent mixture (8); aqueous fraction (9) and organic fraction (10).

Except for the organic fraction, all extracts significantly reduced inflammatory mediation (Figure 5) at a minimum of one tested concentration. The aqueous extract fully reduced NO concentration and was the most effective. The water–acetone (98.31% and 74.74%) and solvent mixture (53.65% and 12.24%) were the only extracts capable of reducing NO production at two concentrations in a dose-dependent manner. Therefore, these extracts were selected for TNF-α, IFN-γ, MCP-1 (Monocyte Chemotactic Protein-1), and IL-6 measurements in RAW264.7 cells. Extracts obtained with acetone, acetonitrile, and ethanol were not evaluated at this step due to the results in antioxidant assays, NO production, and cell viability.

**Figure 6:**
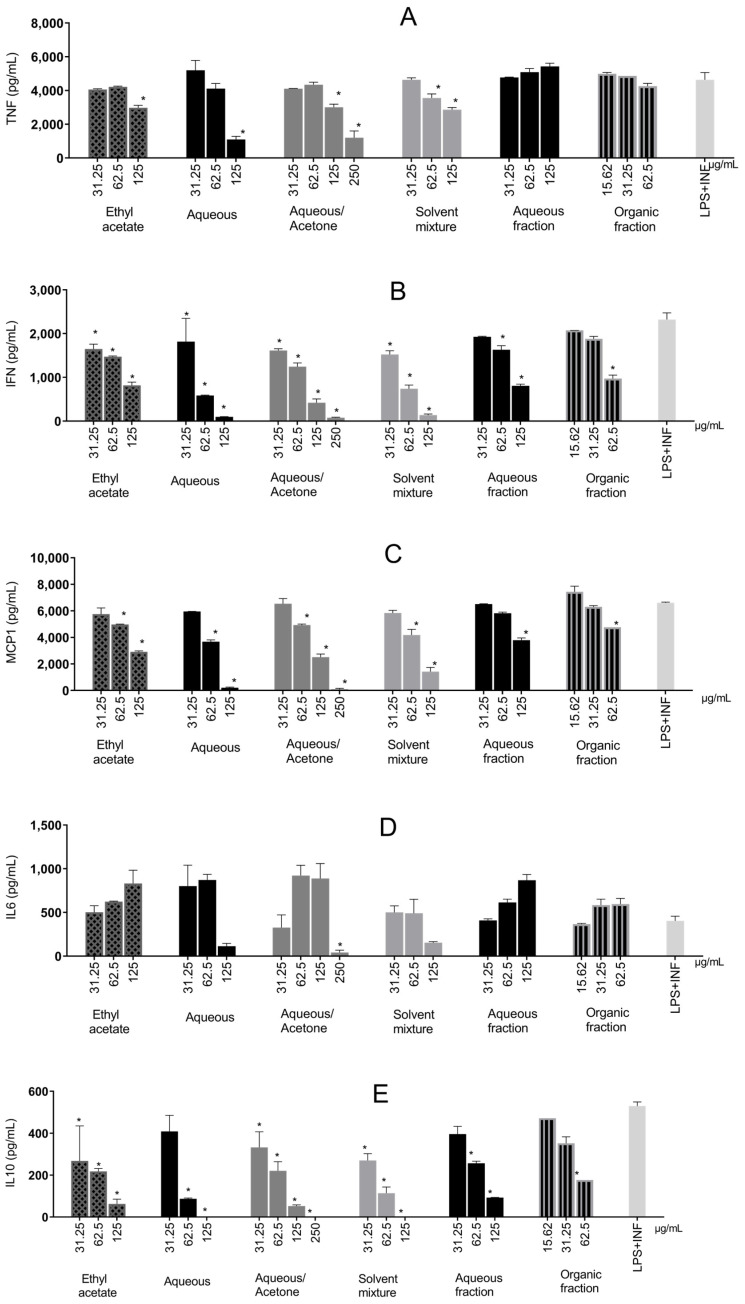
Inflammatory cytokines produced in RAW 264.7 cells stimulated by LPS + IFN-γ and treated with *Stryphnodendron adstringens* stem bark extracts. Values expressed as mean of quadruplicates ± standard deviation. Bars of the same extract with ‘*’ are different from control (LPS + IFN-γ without treatment) by one-way ANOVA followed by Bonferroni post hoc test. * *p* < 0.005. These results are representative of two independent assays. (**A**) Tumor Necrosis Factor-Alpha (TNF-α); (**B**) Interferon-γ (IFN-γ); (**C**) Monocyte Chemoattractant Protein-1 (MCP-1); (**D**) Interleukin-6 (IL-6); (**E**) Interleukin-10 (IL-10).

Except for the aqueous and organic extracts in two steps, all extracts significantly reduced TNF-α production (Figure 6A) compared with the control, displaying a dose–response trend. The aqueous extract reduced cytokine production by 76.39%. Water–acetone reduced TNF-α significantly at two concentrations and was the second-most effective, with a reduction of 74.06%.

Most extracts reduced TNF-α, presumably via NF-κB inhibition. This mechanism has been previously reported for tannins and phenolic groups present in *S. adstringens* [30,38].

All samples significantly reduced IFN-γ production (ranging from 21.61% to 96.7%) compared with the control (Figure 6B). Like TNF-α, water–acetone (96.7%), aqueous (95.89%), and a solvent mixture (94.17%) showed the greatest reductions.

However, unlike TNF-α, IFN-γ production involves the Interferon Regulatory Factor 3 (IRF3). TBK1 (TANK-binding kinase 1) and IKKε are activated upon LPS-TLR4 stimulation, leading to phosphorylation and translocation of IRF3 to the nucleus. IRF3 can also interact with other transcription factors, such as NF-κB, coordinating transcription [39].

Therefore, reports of a synergistic effect between IFN-γ and TNF-α in inflammatory response are not uncommon [40], which can also be observed in the present study, where the samples with the greatest reduction in TNF-α were the ones with the greatest reduction in IFN-γ.

MCP-1 (Figure 6C) was reduced the most by water–acetone (98.72%) and aqueous (96.9%) at the highest concentrations. All samples significantly increased the chemokine, displaying a dose–response trend.

MCP-1 (also known as CCL2) mainly attracts monocytes to inflammatory tissues [41] and is secreted especially by monocytes/macrophages [42]. Binding to CCL2 and its receptor (CCR2) increases monocyte chemotactic protein-1-inducible (MCPIP1) levels and stimulates other inflammatory pathways, such as MAPKs (Mitogen-Activated Protein Kinases), JAK-STAT (Janus kinase-JAK-signal transducer and activator of transcription-STAT), and NF-κB, which drive the production of many inflammatory cytokines [42,43].

Only the water–acetone extract significantly reduced IL-6 production compared with the control (89.62%), as shown in Figure 6D. IL-6 is produced by myeloid cells (neutrophils, monocytes, and macrophages) following TLR stimulation, together with IL-1β and TNF-α, via NF-κB. The latter two can further promote IL-6 production through positive feedback (21). Additionally, IL-6 can self-regulate by binding to its receptor (IL-6R), activating STAT1/STAT3 [44,45], acting as an inflammatory amplifier and interacting synergistically with other pathways [46].

Therefore, extracts that only reduced TNF-α and not IL-6 (aqueous extract, single-phase solvent mixture, and ethyl acetate) probably did not act on STAT3 or inhibit feedback. The exception was water–acetone, which appears capable of acting in this pathway as well.

The water–acetone chromatogram indicates the extraction of hydrophilic, low–low-molecular-weight tannins eluting at the beginning of the run, which are not observed in the chromatograms of other samples. These compounds may cross the cell membrane more readily since they are smaller, potentially accounting for higher anti-inflammatory activity compared with antioxidant activity. A study with *S. adstringens* extracts showed that TNF-α inhibition was higher with polar extracts, while nonpolar extracts exhibited pro-inflammatory activity [3].

EGCG and gallic acid can explain the capacity to inhibit cytokines. There is evidence that EGCG inhibits iNOS, MCP-1, IL-6, IL-1β, IFN-γ, and TNF-α [26]. Gallic acid can suppress NF-κB expression and consequently transcription of inflammatory cytokines [47].

IL-10 quantification, an anti-inflammatory cytokine, yielded results that showed interactions between inflammatory pathways (Figure 6E). There was a decreasing trend in IL-10 production with increasing sample concentration. Aqueous, water–acetone, and solvent mixtures at higher concentrations prevented total IL-10 production.

IL-10 is an important anti-inflammatory interleukin that inhibits inflammatory cytokine production, attenuating the inflammatory activity of immune cells and preventing extensive tissue damage. Anti-inflammatory activity occurs primarily via NF-κB inhibition, reducing the release of cytokines such as TNF-α and IL-1β and adhesion molecules [48].

Thus, the possibility of lower IL-10 production at higher concentrations may reflect inhibition of inflammatory pathways. As demonstrated, IL-10 production is greater at lower concentrations, and it can be observed that IL-10 production is higher at lower sample concentrations. This may occur because greater inflammation at lower concentrations results in the delayed production of IL-10 in an attempt to mitigate inflammatory damage [49].

An herbal medicine or a specific medicine can act in two ways on macrophage inflammatory pathways: attenuating proinflammatory cytokine release into the extracellular medium (e.g., IL-6 and TNF-α), which may already be synthesized and stored in macrophages, and/or affecting cytokine gene expression and thus synthesis and storage [50].

In summary, flavonoids, tannins, and phenolic acids can act by inhibiting phospholipase A2 (PLA2), lipoxygenase (LOX), cyclooxygenase (COX-1 and COX-2), and iNOS, as well as influencing cellular proliferation, angiogenesis, and leukocyte mobilization. Some flavonoids can chelate ions, stabilize radicals, and scavenge them [8,51]; modulate inflammatory cells such as macrophages and pro-inflammatory molecules such as IL-6; and alter the expression of pro-inflammatory genes via transcriptional pathways [51].

In the present study, it became clear that *S. adstringens* extracts attenuate the release of pro-inflammatory cytokines. The inflammatory response in the presence of *S. adstringens* may still occur but could be attenuated, reducing the likelihood of harmful effects.

Considering the experiments performed, extracts obtained with extractive systems composed of water, a solvent mixture, and water–acetone were superior in terms of safety and biological effectiveness, displaying lower cytotoxicity and greater anti-inflammatory activity. Although water–acetone did not appear among the highest antioxidant activities, its effect on reducing inflammatory cytokines was significant.

In analyzing all results, the extract that demonstrated the best overall extractive performance, higher EGCG concentration, shorter solvent evaporation time, lower cost, lower toxicity, and higher antioxidant activity was the aqueous extract. These findings indicate that water can be a promising option to formulate an adequate polyphenol-rich extract at an industrial scale, and the traditional use of *S. adstringens* extract in tea and aqueous infusions represents a safe and effective extraction method.

## 3. Materials and Methods

Solvents, reference chemicals AG, CG, and EGCG, and analytical-grade reagents ethyl acetate, acetone, phosphoric acid, *n*-butanol, dichloromethane, 96% ethanol, absolute ethanol, acetonitrile, ethyl acetate, and isopropanol were purchased from Sigma Aldrich (St. Louis, MO, USA) and Merck (Darmstadt, Germany).

RAW 264.7 macrophage cells were provided by Professor Paula Melo de Abreu Vieira of the Federal University of Ouro Preto. Folin-Ciocalteau reagents, gallic acid, Trolox, TPTZ (2,4,6-tri(2-pyridyl)-s-triazine), AAPH (2,2′-azobis(2-methylpropionamidine) dihydrochloride), flow cytometry buffers, RPMI1640 culture medium, sulforhodamine B, LPS, and IFN were purchased from Sigma-Aldrich (St. Louis, MO, USA). Fetal bovine serum was obtained from Cultilab (Campinas, Brazil). BD™ Cytometric Bead Array (CBA) kits were purchased from BD Biosciences Pharmingen (San Diego, CA, USA). Analytical-grade reagents were also used.

### 3.1. Plant Material

The collection of *Stryphnodendron adstringens* (Martius) Coville (Fabaceae) was carried out in Santa Cruz de Minas and Ritápolis in Minas Gerais. The samples were identified by Professor Dr. João Renato Stehmann of the Department of Botany of the Institute of Biological Sciences of UFMG (Federal University of Minas Gerais State). The plant exsiccates were deposited in the Herbarium of the Department of Botany of the Institute of Biological Sciences of UFMG under number BHCB111231. The provisions of the National System for the Management of Genetic Heritage and Associated Traditional Knowledge were with protocol number AB46C9A.

### 3.2. S. adstringens Extracts

#### 3.2.1. *S. adstringens* Extracts in One Step

**Figure 7:**
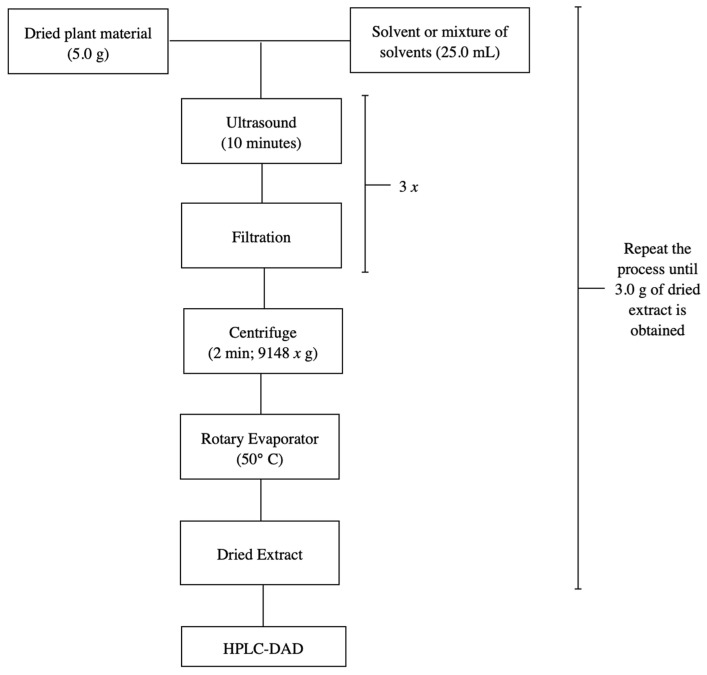
Flowchart illustrating the one-step *S. adstringens* extraction process employing different solvents or solvent mixtures.

In previous studies, various extraction methodologies were investigated targeting two objectives: the development of the analytical method and the large-scale production of extracts for the characterization of the chemical matrix and pharmacological evaluation. The methodologies that demonstrated the best performance in terms of speed and efficiency were ultrasound-assisted maceration and percolation, respectively. Therefore, the same were reproduced in this study [18]. The stem bark of *S. adstringens* was crushed and pulverized in a knife mill. Next, 5.0 g of the dried and pulverized plant material was macerated with 25.0 mL of different solvents for 10 min using ultrasound, followed by filtration to retain larger particles to obtain the plant extracts. The process was repeated twice more. The extracts obtained were then combined, centrifuged for 2 min at 9148 g, and the supernatant was evaporated in a rotary evaporator at a water bath temperature of 50 °C until the solvent was completely eliminated, thus obtaining dry crude ethanolic extracts. Figure 7 shows the flowchart of the extraction process for *S. adstringens* carried out in a single step using the following solvents or solvent mixture: ethyl acetate, acetone, acetonitrile, water, dichloromethane, ethanol, water:acetone mixture (1:1), water:ethanol mixture (1:1) and ethyl acetate:isopropanol:*n*-butanol mixture (0.7:0.2:0.1).

#### 3.2.2. *S. adstringens* Extracts in Two Steps

**Figure 8:**
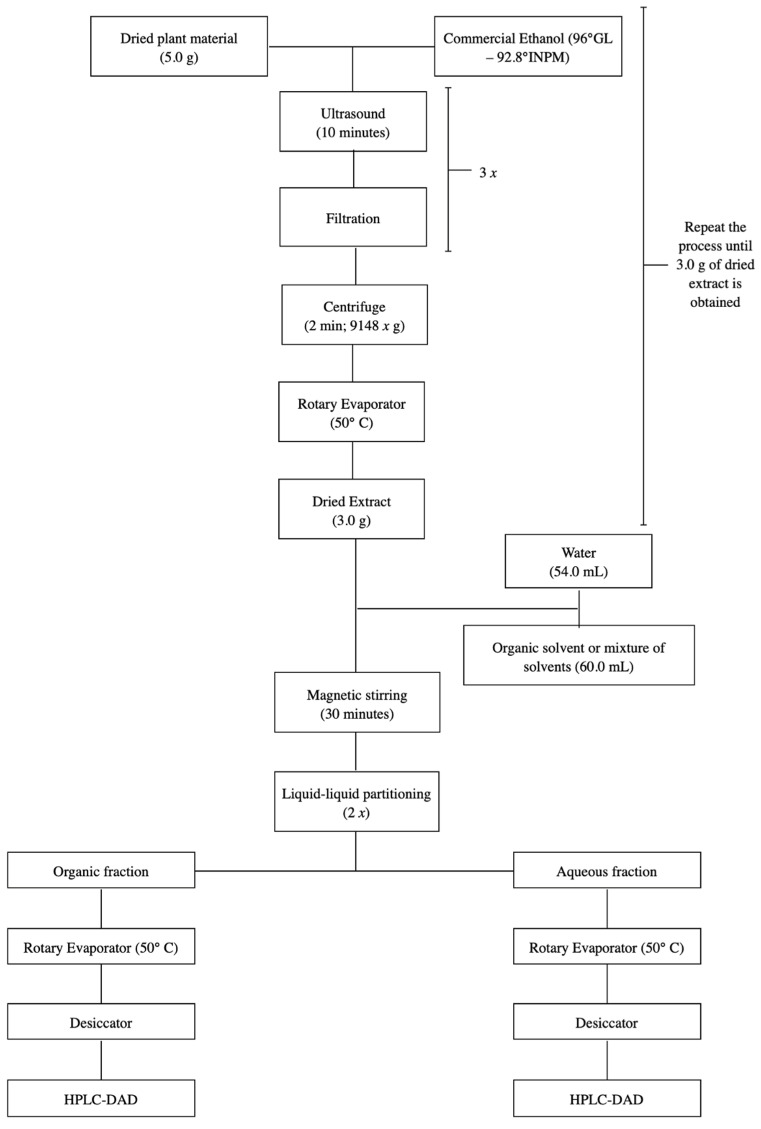
Flowchart illustrating the two-step *S. adstringens* extraction process employing different solvents or solvent mixtures.

The ethanolic extract of *S. adstringens* was subjected to extraction by partition between immiscible solvents. A 3.0 g portion of the ethanolic extract was resuspended in 54 mL of water under magnetic stirring for 30 min, and subjected to partition with the solvent mixture:ethyl acetate:isopropanol:*n*-butanol (42:12:6) 54 mL (1:1 extraction) [18]. The extraction was repeated twice more and the organic and aqueous phases were concentrated into residue in a rotary evaporator and kept in a desiccator until complete elimination of the solvent, resulting in the organic and aqueous extracts. This process was repeated for accumulation (Figure 8).

### 3.3. Analysis of S. adstringens Extracts by HPLC-DAD

#### 3.3.1. Sample and Standard Preparation

For chromatographic analyses, sample preparation for chromatographic analysis was performed as described in Section 3.2.2. 5 mg/mL solutions of the organic phase were prepared in HPLC-grade methanol/ultrapure water (1:9). The prepared solutions were subsequently centrifuged for 10 min at 9184 g and filtered through 0.45 µm PTFE membranes for injection into the HPLC system. Chemical reference substances to prepare the standard were weighed to the nearest 5 mg of epigallocatechin gallate (EGCG; minimum 95%) purchased from Sigma (Milwaukee, WI, USA) and transferred to 5 mL volumetric flasks. Methanol was added to reach a volume of 5 mL, obtaining a methanolic solution of EGCG with a concentration of 1 mg/mL.

#### 3.3.2. Analytical Conditions

The chromatographic profiles of *S. adstringens* extracts were obtained on a Shimadzu LC-20A Prominence chromatograph coupled with a photodiode array detector (Shimadzu SPD20A). A LiChrospher 100 RP-18 reversed-phase column (250 × 4 mm i.d., 5 µm) was used according to the method developed and validated by [18]. The degassed mobile phase consisted of aqueous 0.1% phosphoric acid (solution A) and 0.1% phosphoric acid in acetonitrile (solution B). The linear gradient used was from A-B (95:5 [*v*/*v*]) to A-B (60:40 [*v*/*v*]) in 60 min, which was followed by cleaning and reconditioning of the column in 15 min, with a flow rate of 1 mL/min and a temperature of 40 °C. Detection was performed by UV-DAD, with chromatograms extracted at a wavelength of 210 nm, and data acquisition was conducted using Shimadzu Lab Solutions software (Version 5.57 SP1). In turn, 10 µL aliquots were automatically used in the HPLC system for injection of extracts and EGCG. This method was developed and validated for quantifying the chemical marker epigallocatechin gallate (EGCG), with the line equation used for quantification being: y = 10.8255x − 23.16 [18].

### 3.4. Antioxidant Activity 

In order to determine the antioxidant activity of different *S. adstringens* extracts and correlate the one that best merges antioxidant potential with the principles of green chemistry, we chose methods that evaluate this activity through distinct mechanisms. Furthermore, we also considered methods that assessed this activity under different pH and temperature conditions. These methods are accessible, fast, and typically automated, being used predominantly in screening and initial assessment of new antioxidant extracts [24].

The dried extracts and present compounds were solubilized with 1% dimethyl sulfoxide (DMSO), and the volume was completed with Milli-Q water until reaching a concentration of 1 mg/mL. The exception was the extract obtained with dichloromethane, which used 2% DMSO. Then, the volume was completed to the mentioned concentration, centrifuged for 10 min at 3000 revolutions per minute (rpm), and the supernatant was collected and used in the experiments described below.

#### 3.4.1. Folin–Ciocalteau Reagent Reducing Substances (FCRRS)

The assay was performed in triplicate for each extract following Medina (2011) [52]. First, 450 μL of distilled water and 50 μL of extract, gallic acid standard solutions (50, 100, 200, 300, 400, 500, 600 μg/mL), or distilled water for blank were added and mixed. Then, the Folin–Ciocalteu reagent (50 μL) was added and mixed. Next, 500 μL of 7% Na_2_CO_3_ (sodium carbonate) and 200 μL of distilled water were added and mixed. The mixture was subsequently allowed to react at room temperature in the dark for 90 min. The absorbance was measured at 765 nm using an Epoch™ Microplate Spectrophotometer (BioTek, Winooski, VT, USA), and the results were expressed as mg gallic acid equivalents (GAE) per gram of sample (mg GAE/g sample).

#### 3.4.2. Ferric Reducing Antioxidant Power (FRAP)

The FRAP assay followed the protocol available from Embrapa (2006) [53]. First, 2.5 mL of 20 mM ferric chloride solution (FeCl3), 2. 5 mL of 10 mM TPTZ solution and 25 mL of 0.3 M acetate buffer were added for each 30 mL of FRAP reagent. Next, 30 μL of extract, standard (100; 200; 400; 800; 1200 and 1600 μM) or blank was mixed with 90 μL of water and 900 μL of FRAP reagent in the dark. The mixture was then incubated at 37 °C for 30 min. The absorbance was measured at 595 nm in an Epoch™ Microplate Spectrophotometer (BioTek, Winooski, VT, USA), and the results were expressed as mg Trolox equivalent (TE) per gram of sample (mg TE/g sample).

#### 3.4.3. Oxygen Radical Absorbance Capacity (ORAC)

The ORAC assay was performed according to Ou et al. (2002) and Dávalos et al. (2004) [54]. Thus, 20 μL of the extract or standard (Trolox 25; 50; 100; 300; 500 and 700 μM), 120 μL of sodium fluorescein in potassium phosphate buffer (pH 7.4) (final concentration 0.378 μg/mL) and 60 μL of AAPH (108 mg/mL) were used to perform the oxygen radical absorbance capacity (ORAC) assay. Fluorescence was then measured every minute for 80 min with an excitation wavelength of 485 nm and an emission wavelength of 520 nm in a PerkinElmer Victor X3 Multi-Mode Microplate Reader (Waltham, MA, USA). The antioxidant capacity was expressed as μmol Trolox equivalent (TE) per gram of sample (μmol TE/g sample), based on the area under the curve (AUC) for the decline in the fluorescence time, following the Equation (1):

AUC = 1 + f2/f1 + f3/f1 + f4/f1 + fn/f1(1)

In which:

f1 = fluorescence reading at time 1

f2 = fluorescence reading at time 2

fn = fluorescence reading at time 80

### 3.5. In Vitro Cell-Based Assays

#### 3.5.1. Cell Culture

The murine macrophage cell line RAW 264.7 was used to conduct a viability assay and to assess production of inflammatory mediators. The cells were cultured in RPMI 1640 medium supplemented with 10% fetal bovine serum at 37 °C in 5% CO_2_.

#### 3.5.2. Cell Viability Assay

The cell viability assay was assessed by SRB method. Cells were seeded into 96-well microplates at a density of 5 × 10^5^ cells/mL and incubated for 24 h. After removing the supernatants, cells were incubated with extracts (1000; 500; 250; 125; 62.5; 31.25; 15.62; 7.81; 3.90; 1.95 and 0.97 µg/mL) for 24 h. The supernatants were removed, and the wells were washed twice with PBS 1*x*. The plates were incubated in the refrigerator for 1 h with Trichloroacetic acid 20% to fix the cells. Then, the plates were washed five times and 40 uL of sulforhodamine B was added. After 30 min, the plate was washed with acetic acid and 200 uL of Tris was added. The optical density was measured at 490 nm in an Epoch™ Microplate Spectrophotometer (BioTek, Winooski, VT, USA). The results were expressed as relative cell viability (%) using the blank treatment (RPMI 1640 complete medium) as control, adopting 70% viability as the cut-off point [36].

#### 3.5.3. Production of Inflammatory Mediators

Murine cells were seeded in a 96-well culture plate at a density of 5 × 10^5^ cells/mL and incubated for 24 h at 37 °C. After removal of the supernatants, the cells were treated with the extracts at different concentrations (100 μL) within the non-toxic range determined in viability experiments, between 250 μg/mL and 15.6 μg/mL. Cells were incubated in culture medium without extract or inflammatory stimulus for the control. After distribution of all extracts, the inflammatory stimuli lipopolysaccharide (LPS) (5 μg/mL) and IFN-γ (20 ng/mL) were added (100 μL, 1:1 ratio). The plates were incubated for 24 h, and the supernatants were stored at −80 °C for subsequent determination of inflammatory cytokines and nitric oxide. Experiments were performed in triplicate.

#### 3.5.4. Determination of Nitrite Production

Nitric oxide (NO) released into the supernatants of the cell cultures was indirectly determined using a quantitative colorimetric assay based on Griess reaction (1% sulphanilamide, 2.5% phosphoric acid, and 0.1% naphthylethylenediamine). Next, 50 μL of supernatant was mixed with 50 μL of Griess reagent in a 96-well microplate. The optical density was measured at 540 nm in an Epoch™ Microplate Spectrophotometer (BioTek, Winooski, VT, USA), and the concentration of nitrite in the samples was determined by comparison with a standard curve of sodium nitrite (0.5; 0.25; 0.125; 0.625; 0.312; 0.156; 0.0078; 0.0039; 0.0019; 0.00097; 0.000483 and 0.00024 nmol/L). Untreated and LPS-stimulated cells were used as the blank control. Two independent experiments were performed in duplicate.

#### 3.5.5. Determination of Inflammatory Cytokines

The Interleukin-6 (IL-6), Interleukin-10 (IL-10), Monocyte Chemoattractant Protein-1 (MCP-1), Interferon-γ (IFN-γ), and Tumor Necrosis Factor (TNF) levels were measured in macrophage supernatants according to the manufacturer’s instructions of BD™ Cytometric Bead Array (CBA) (BD Biosciences Pharmingen, San Diego, CA, USA). Untreated and stimulated cells were used as the blank control.

### 3.6. Statistical Analysis

The statistical analysis was performed using the GraphPad Prism version 9.0 program (GraphPad Software, San Diego, CA, USA). Data were assessed for normality with the D’Agostino-Pearson test. Statistical differences were determined by one-way ANOVA, followed by Tukey’s post hoc test for multiple comparisons or Bonferroni for comparisons with the control. Variables were described by mean and standard deviation. The significance level was set at *p* = 0.05 to achieve 95% confidence between comparisons.

## 4. Conclusions

This study demonstrated the possibility of optimizing the extraction process from *S. adstringens* (barbatimão) stem bark, reducing it from two steps to just one, without compromising the chemical and biological efficiency of the extracts. Comparative analysis revealed that single-step extraction systems, especially those based on water, ethanol, water-acetone, and water-ethanol, offer equivalent or superior performance in terms of yield, phytochemical marker concentration, evaporation time, cost, toxicity, and feasibility for industrial scale-up.

From a chemical perspective, the extracts obtained exhibited similar profiles, rich in gallic acid and condensed tannins, with emphasis on epigallocatechin gallate (EGCG), a compound associated with antioxidant and anti-inflammatory activity. Although the organic extract obtained in two steps presented higher EGCG concentrations, its production required a larger amount of raw material, the use of toxic solvents, and a longer preparation time, making its application on an industrial scale unfeasible.

All extracts showed low cytotoxicity and significant antioxidant and anti-inflammatory activity in biological assays, with emphasis on the aqueous, water-acetone, and one-step solvent mixture systems, which significantly reduced inflammatory mediators such as NO, TNF-α, IFN-γ, MCP-1, and IL-6. Among these, the aqueous extract demonstrated the best balance between safety, cost, practicality, and efficacy, confirming the relevance of its traditional use in infusions and reinforcing its potential as a raw material for safe and sustainable herbal formulations.

Thus, the results obtained herein demonstrate that the use of water as an extraction solvent aligns with the principles of green chemistry, reduces the generation of toxic waste, ensures greater operational safety, and represents a viable and effective alternative for the development of industrial products based on *S. adstringens*, combining tradition, science, and sustainability.

## Figures and Tables

**Table 1:** Evaluation results of extracts obtained from the stem bark of *Stryphnodendron adstringens*.

Number of Steps		Solvents and Solvent Mixtures	Mass (g) of Initial Plant Material	Yield (mg Dry Extract)	Yield (mg Dry Extract/g Initial Plant Material)	EGCG Concentration (µg/mL)	EGCG Yield (mg/g Dry Extract)	Time (min) for Solvent Elimination ^1^	Acute Tox. ^2^	Cost/L Pure Substance (US$)	Controlby FP ^3^	Folin	FRAP	ORAC
One step	1	Ethyl acetate	5.0	17.8	3.56	177.33 ± 10.11	0.63 ± 0.04	5	4100	11.05	S	347.43	3250.11	3585.49
	2	Acetone	5.0	10.0	2.00	81.88 ± 1.93	0.17 ± 0.01	0	5000	9.68	S	499.17	4798	3885.92
	3	Acetonitrile	5.0	37.8	7.56	181.84 ± 31.79	1.33 ± 0.20	5	2460	25.37	S	473.77	4123.67	6809.51
	4	Water	5.0	905.6	181.12	25.26 ± 6.37	4.58 ± 1.15	110	0	0.02	N	589.46	3912.56	2842.89
	5	Dichloromethane	5.0	29.6	5.92	13.05 ± 1.71	0.08 ± 0.01	0	1600	12.05	S	141.043	783.443	633.067
	6	Ethanol	5.0	588.0	117.60	63.87 ± 1.38	7.51 ± 0.17	2	5000	1.39	N	444.57	4687.89	2604.59
	7	Water:Acetone (1:1)	5.0	755.2	151.04	45.60 ± 2.18	6.89 ± 0.33	50	10,000	4.84	S	358.22	3912.56	2377.51
	8	Water:Ethanol (1:1)	5.0	3355.2	671.04	35.68 ± 13.67	2.39 ± 0.91	50	10,000	0.70	N	436.68	4289.11	2787.15
	9	Solvent mixture Ethyl acetate:Isopropanol:*n*-butanol (0.7:0.2:0.1)	5.0	281.3	56.26	84.02 ± 3.06	4.73 ± 0.17	50	3670	11.71	S	602.67	5226.89	3203.07
Two steps	10	Solvent mixture-FA (aqueous phase) Ethyl acetate:Isopropanol:*n*-butanol (0.7:0.2:0.1)	25.0	195.0	7.80	56.64 ± 11.96	0.52 ± 0.03	50	3670	13.11	S	518.47	4851.62	1726.95
	11	Solvent mixture-FO (organic phase) Ethyl acetate:Isopropanol:*n*-butanol (0.7:0.2:0.1)	25.0	645.5	25.82	79.54 ± 27.06	2.01 ± 0.73	50	3670	13.11	S	503.35	4202.1	2771.64

^1^ Time elapsed between the beginning and the end of the complete solvent evaporation process in the rotary evaporator. The solvent volumes were 75 mL for the one-step method and 162 mL for the two-step method. ^2^ Oral LD_50_ values in mg/kg of mouse are presented in the MSDS of each product. ^3^ The lists of chemical products controlled by the Federal Police are provided in Ordinance No. 1274 of 25 August 2003.

## Data Availability

The original contributions presented in this study are included in the article. Further inquiries can be directed to the corresponding author.

## Supplementary Materials

The following supporting information can be downloaded at: https://www.mdpi.com/article/10.3390/molecules31020224/s1, Figure S1: Chromatographic profile of the *Stryphnodendron adstringens* stem bark extract obtained by a one-step/two-step extraction process, containing the majority of the identified peaks (GA, gallic acid; GC, gallocatechin; EGC, epigallocatechin; C, catechin; EGCG, epigallocatechin gallate; MGC, 4′-O-Methylgallocatechin); (a) ethyl acetate (1), (b) acetone (2), (c) acetonitrile (3), (d) water (4) (e) dichloromethane (5), (f) ethanol (6), (g) water:acetone (1:1) (7), (h) water:ethanol (1:1) (8) and (i) one step ethyl acetate:isopropanol:*n*-butanol (0.7:0.2:0.1) (9), (j) two-steps ethyl acetate:isopropanol:*n*-butanol (0.7:0.2:0.1)-water phase (10) (k) two-step ethyl acetate:isopropanol:*n*-butanol (0.7:0.2:0.1)-organic phase (11). Conditions: pre-column C18 (XDB Zorbax^®^, 4 × 4 mm I.D.; 5 μm), attached to a C18 column (LiChrospher100, 250 × 4 mm I.D.; 5 μm); linear gradient elution, A–B (95:5% [*v*/*v*]) to A–B (60:40% [*v*/*v*]) in 60 min with 0.1% phosphoric acid (A) and 0.1% phosphoric acid in acetonitrile (B); 1 mL/min; 4 °C; 10 mL; l = 210 nm.

## Abbreviations

The following abbreviations are used in this manuscript:

ANOVA	Analysis of Variance
ARE	Antioxidant Response Element
AUC	Area Under the Curve
C	Catechin
COX	Cyclooxygenase
CRS	Chemical Reference Substance
DMSO	Dimethyl Sulfoxide
EGC	Epigallocatechin
EGCG	Epigallocatechin Gallate
ESI-MS	Electrospray Ionization Mass Spectrometry
FCRRS	Folin–Ciocalteau Reagent Reducing Substances
FRAP	Ferric Reducing Antioxidant Power
GA	Gallic Acid
GC	Gallocatechin
HPLC-DAD	High-Performance Liquid Chromatography (HPLC) coupled to a Diode Array Detector (DAD)
IFN-γ	Interferon-Gamma
IKKε	Inhibitor of Nuclear Factor Kappa-B Kinase-ε
IL-1β	Interleukin 1 Beta
IL-6	Interleukin 6
IL-10	Interleukin 10
iNOS	Inducible Nitric Oxide Synthase
IRF3	Interferon Regulatory Factor
JAK-STAT	Janus Kinase (JAK)-Signal Transducer and Activator of Transcription (STAT)
Keap1	Protein Encoded by the Keap1 Gene
LD50	Median Lethal Dose
LOX	Lipoxygenase
LPS	Lipopolysaccharide
MAPKs	Mitogen-Activated Protein Kinases
MCP-1	Monocyte Chemotactic Protein-1
MGC	4′-O-Methylgallocatechin
Na_2_CO_3_	Sodium Carbonate
NFκB	Nuclear Factor Kappa B
NO	Nitric Oxide
Nrf2	Nuclear Factor Erythroid 2
ORAC	Oxygen Radical Absorbance Capacity
PLA2	Phospholipase A2
ROSs	Espécies Reativas de Oxigênio
SUS	Unified Health System
TBK1	TANK-Binding Kinase 1
TE	Trolox equivalent
TLR-4	Toll-Like Receptor
TNF-α	Tumor Necrosis Factor Alpha
TPTZ	Tripyridyltriazine
UPLC	Ultra Performance Liquid Chromatography
