# Supplementary material for: Optimization of Stryphnodendron adstringens (Barbatimão) Extraction: Chemical Evaluation, Cytotoxicity, Antioxidant and Anti-Inflammatory Activities
Source: Molecules. 2026 Jan 9;31(2):224. doi: 10.3390/molecules31020224 (PMC12843935; doi:10.3390/molecules31020224)
Supplement: Supplementary file 1 [file molecules-31-00224-s001.zip › molecules-4009121-supplementary.pdf]

Supplementary Materials: The following supporting information can be downloaded at: https://www.mdpi.com/article/doi/s1, Figure S1: Chromatographic profile of the *Stryphnodendron adstringens* stem bark extract obtained by a one-step/two-step extraction process, containing the majority of the identified peaks (GA, gallic acid; GC, gallocatechin; EGC, epigallocatechin; C, catechin; EGCG, epigallocatechin gallate; MGC, 4'-O-Methylgallocatechin); a) ethyl acetate (1 in Table 1), b) acetone (2 in Table 1), c) acetonitrile (3 in Table 1), d) water (4 in Table 1) e) dichloromethane (5 in Table 1), f) ethanol (6 in Table 1), g) water:acetone (1:1) (7 in Table 1), h) water:ethanol (1:1) (8 in Table 1) and i) one step ethyl acetate:isopropanol:*n*-butanol (0.7:0.2:0.1) (9 in Table 1), j) two-steps ethyl acetate:isopropanol:*n*-butanol (0.7:0.2:0.1) - water phase (10 in Table 1) k) two-step ethyl acetate:isopropanol:*n*-butanol (0.7:0.2:0.1) - organic phase (11). Conditions: pre-column C18 (XDB Zorbax®, 4 × 4 mm I.D.; 5 µm), attached to a C18 column (LiChrospher100, 250 × 4 mm I.D.; 5 µm); linear gradient elution, A–B (95:5% [v/v]) to A–B (60:40% [v/v]) in 60 min with 0.1% phosphoric acid (A) and 0.1% phosphoric acid in acetonitrile (B); 1 mL/min; 4°C; 10ml; l = 210 nm.

a)

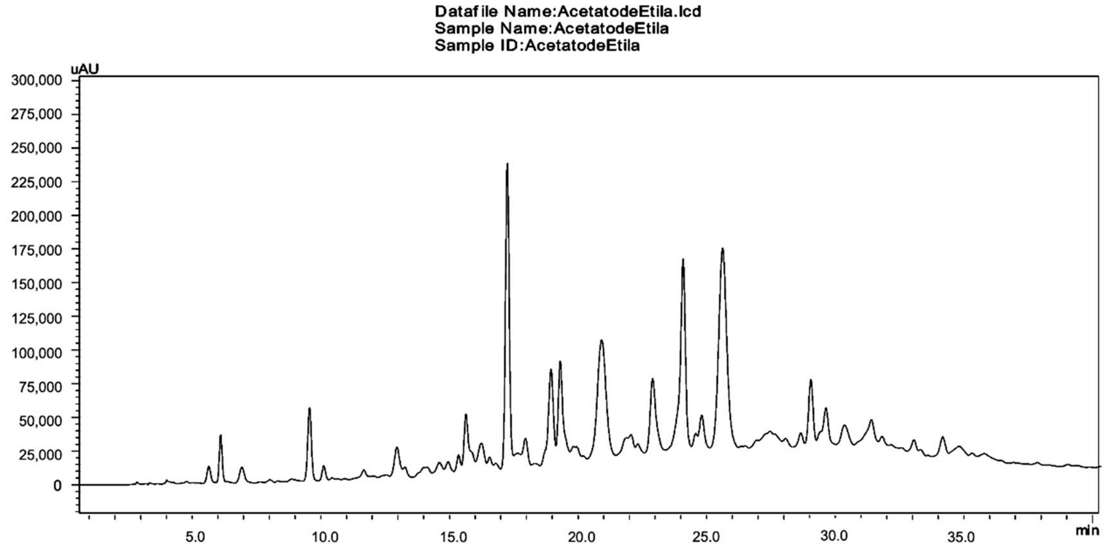

b)

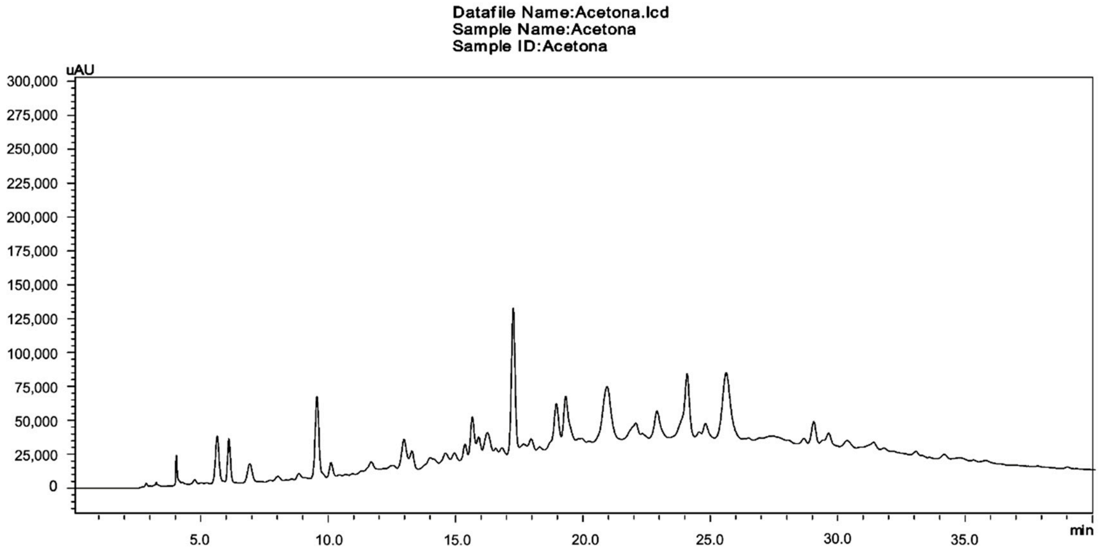
Datafile Name:Acetona.lcd
Sample Name:Acetona
Sample ID:Acetona
uAU
300,000
275,000
250,000
225,000
200,000
175,000
150,000
125,000
100,000
75,000
50,000
25,000
0
5.0
10.0
15.0
20.0
25.0
30.0
35.0
min

c)

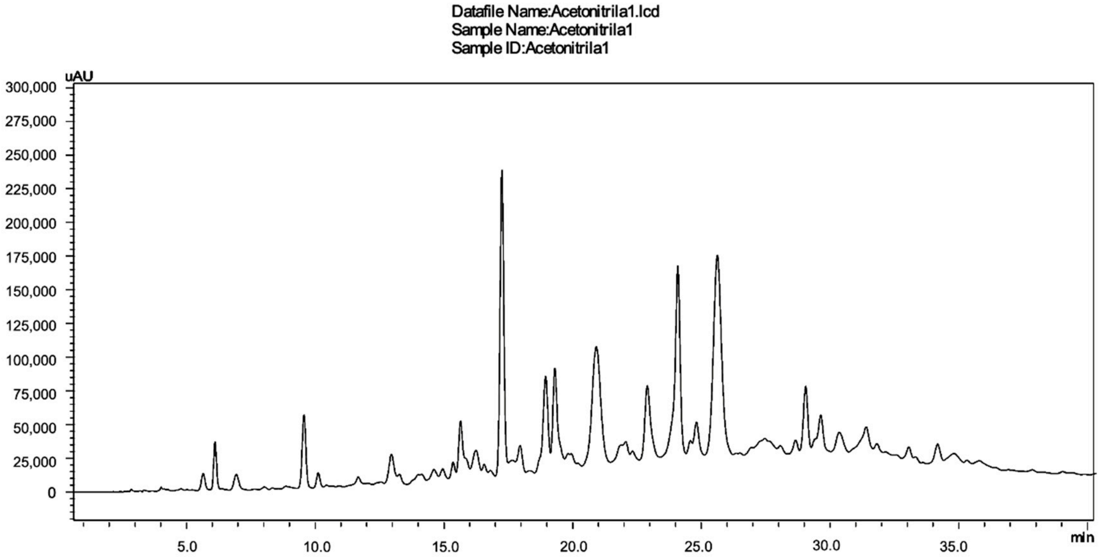
Datafile Name:Acetonitrila1.lcd
Sample Name:Acetonitrila1
Sample ID:Acetonitrila1
uAU
300,000
275,000
250,000
225,000
200,000
175,000
150,000
125,000
100,000
75,000
50,000
25,000
0
5.0
10.0
15.0
20.0
25.0
30.0
35.0
min

d)

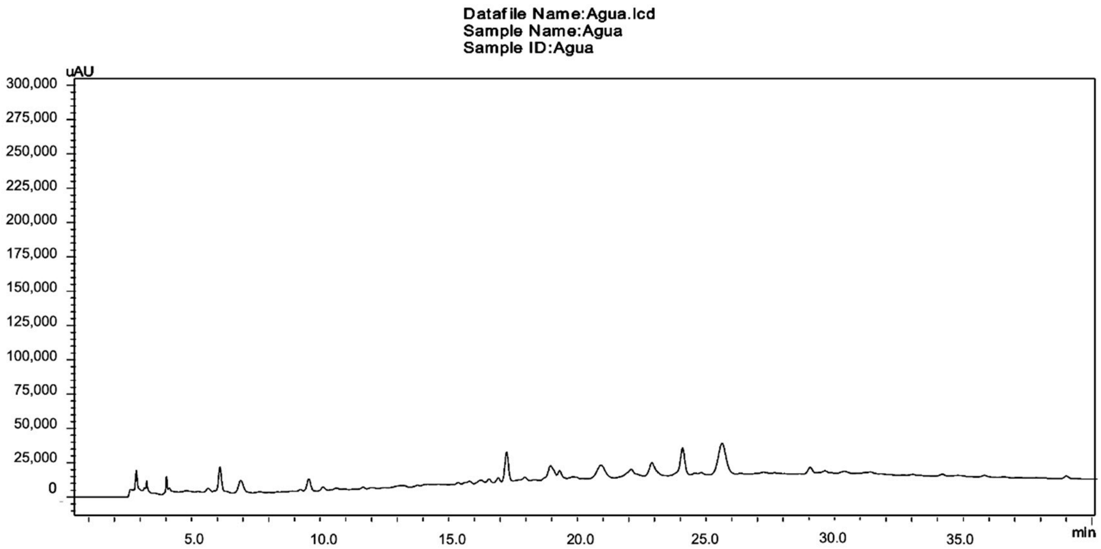
Datafile Name:Agua.lcd
Sample Name:Agua
Sample ID:Agua
uAU
300,000
275,000
250,000
225,000
200,000
175,000
150,000
125,000
100,000
75,000
50,000
25,000
0
5.0
10.0
15.0
20.0
25.0
30.0
35.0
min

e)

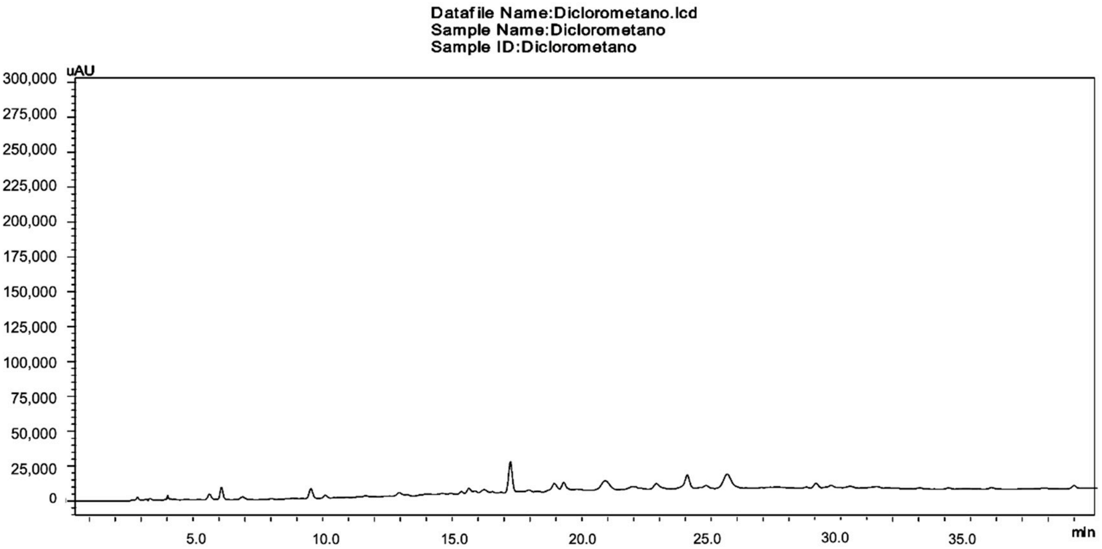
Datafile Name:Diclorometano.lcd
Sample Name:Diclorometano
Sample ID:Diclorometano
uAU
300,000
275,000
250,000
225,000
200,000
175,000
150,000
125,000
100,000
75,000
50,000
25,000
0
5.0
10.0
15.0
20.0
25.0
30.0
35.0
min

f)

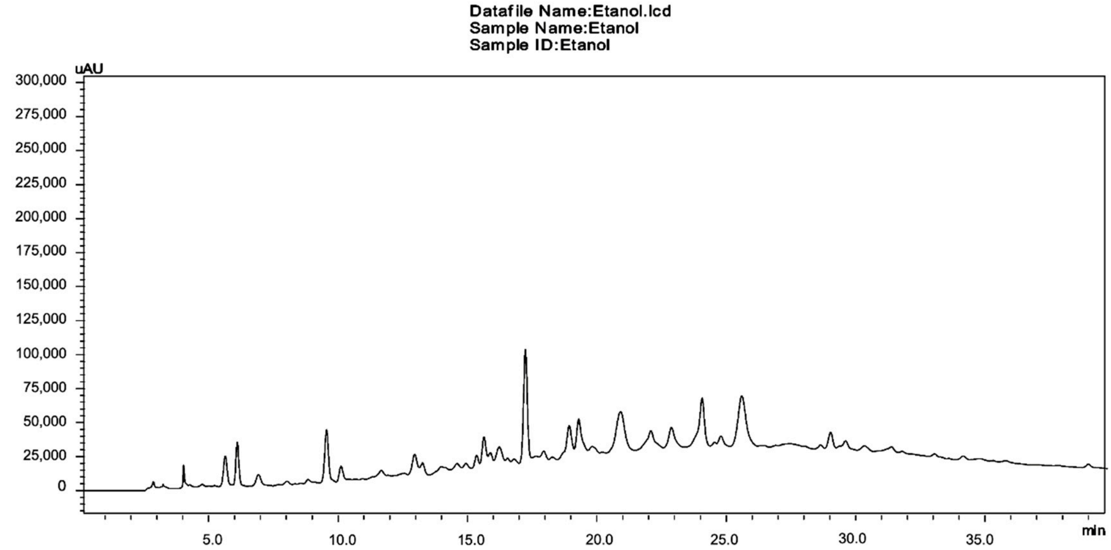
Datafile Name:Etanol.lcd
Sample Name:Etanol
Sample ID:Etanol
uAU
300,000
275,000
250,000
225,000
200,000
175,000
150,000
125,000
100,000
75,000
50,000
25,000
0
5.0
10.0
15.0
20.0
25.0
30.0
35.0
min

g)

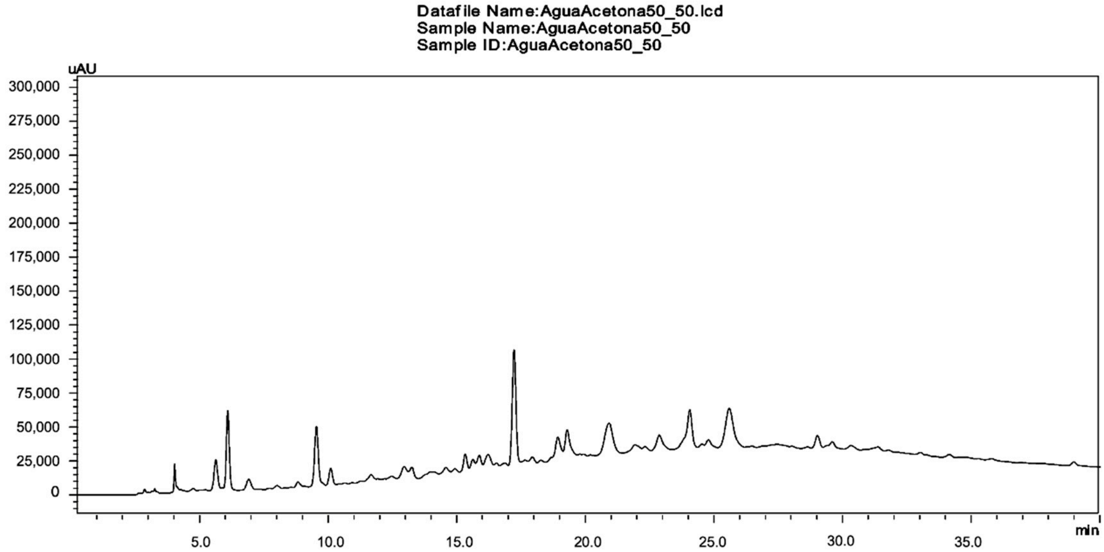
Datafile Name:AguaAcetona50_50.lcd
Sample Name:AguaAcetona50_50
Sample ID:AguaAcetona50_50
uAU
300,000
275,000
250,000
225,000
200,000
175,000
150,000
125,000
100,000
75,000
50,000
25,000
0
5.0
10.0
15.0
20.0
25.0
30.0
35.0
min

h)

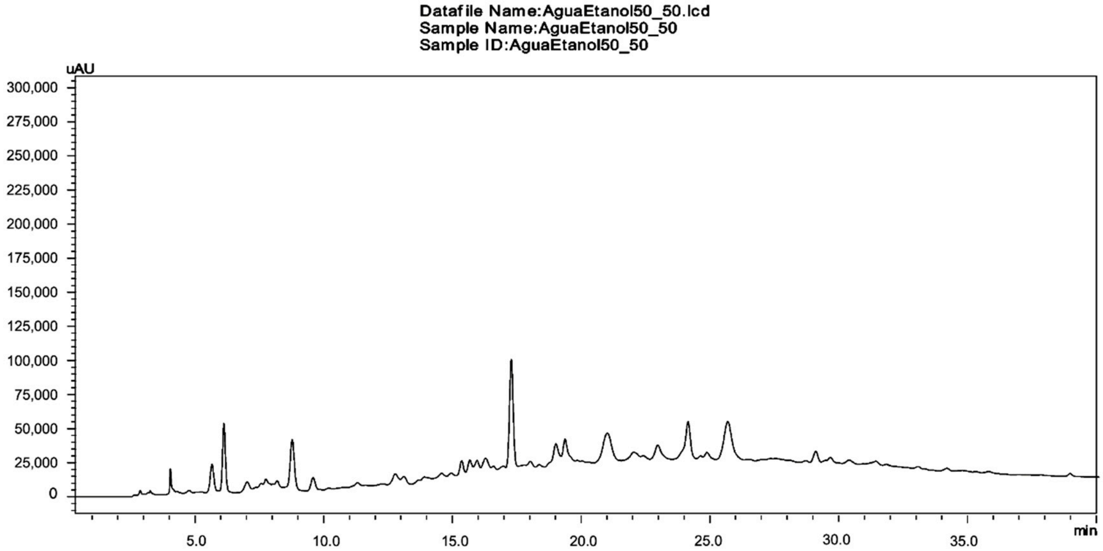
Datafile Name:AguaEtanol50_50.lcd
Sample Name:AguaEtanol50_50
Sample ID:AguaEtanol50_50
uAU
300,000
275,000
250,000
225,000
200,000
175,000
150,000
125,000
100,000
75,000
50,000
25,000
0
5.0
10.0
15.0
20.0
25.0
30.0
35.0
min

i)

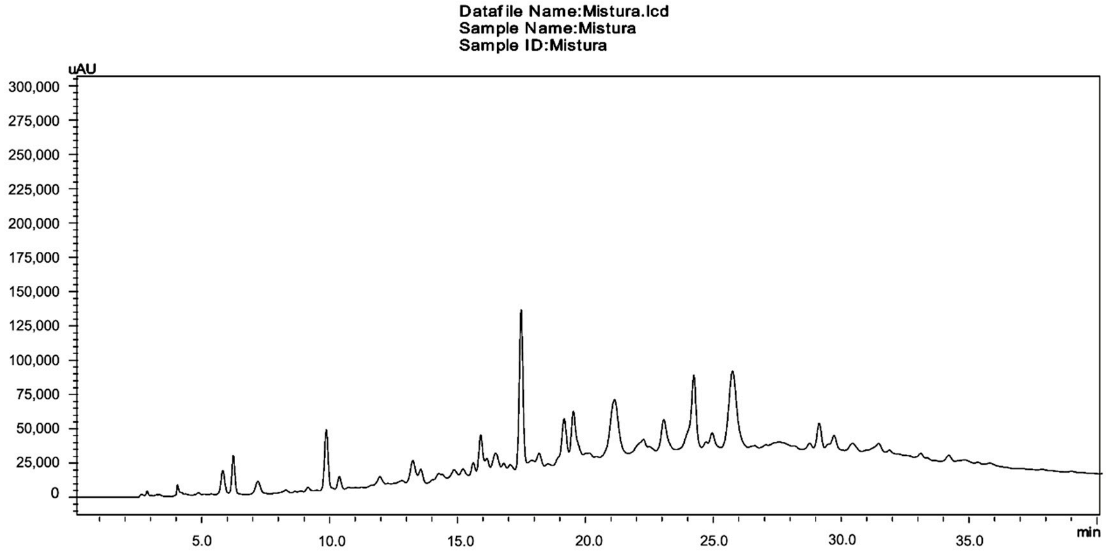
Datafile Name:Mistura.lcd
Sample Name:Mistura
Sample ID:Mistura
uAU
300,000
275,000
250,000
225,000
200,000
175,000
150,000
125,000
100,000
75,000
50,000
25,000
0
5.0
10.0
15.0
20.0
25.0
30.0
35.0
min

J)

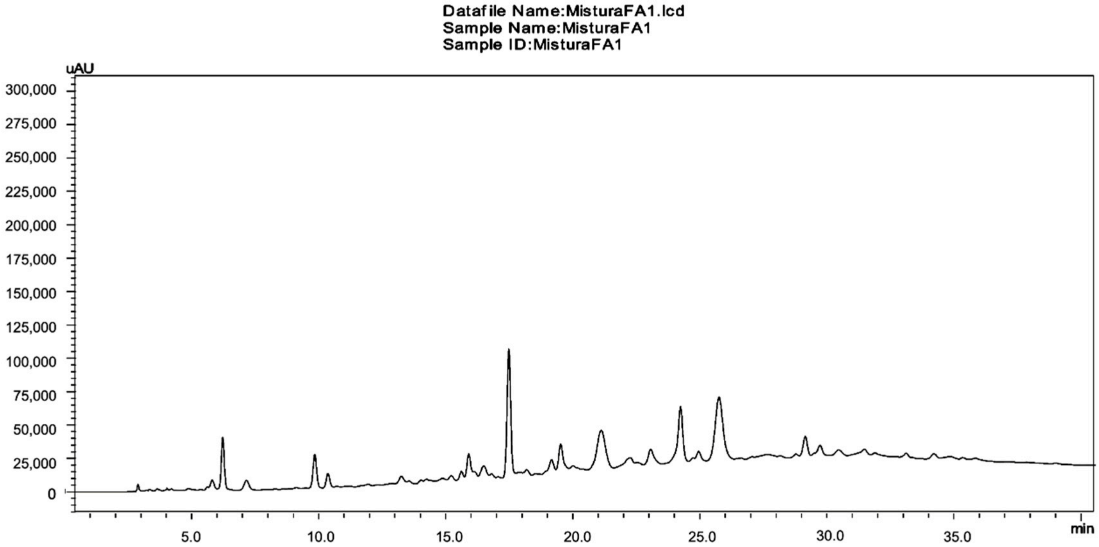
Datafile Name:MisturaFA1.lcd
Sample Name:MisturaFA1
Sample ID:MisturaFA1
uAU
300,000
275,000
250,000
225,000
200,000
175,000
150,000
125,000
100,000
75,000
50,000
25,000
0
5.0
10.0
15.0
20.0
25.0
30.0
35.0
min

l)

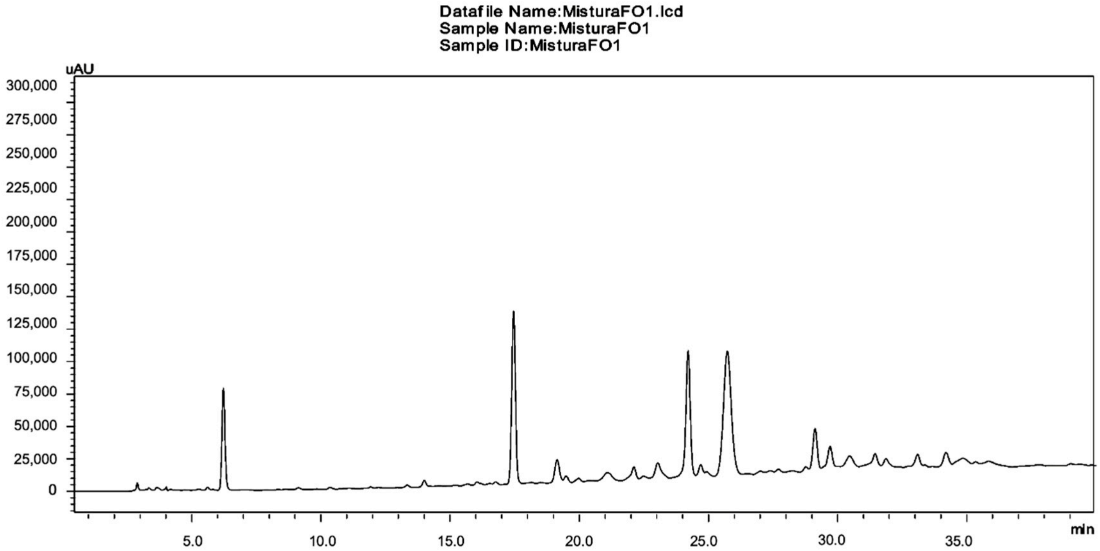
Datafile Name:MisturaFO1.lcd
Sample Name:MisturaFO1
Sample ID:MisturaFO1
uAU
300,000
275,000
250,000
225,000
200,000
175,000
150,000
125,000
100,000
75,000
50,000
25,000
0
5.0
10.0
15.0
20.0
25.0
30.0
35.0
min
